# A Rare Presentation of Bilateral Pheochromocytoma With a Flank Pain Radiating to the Back: A Case Report and Narrative Review

**DOI:** 10.1016/j.aed.2025.09.014

**Published:** 2025-09-30

**Authors:** Nadia Nadeem, Nauman Zafar, Sarmad Imtiaz

**Affiliations:** 1 Department of Hematology and Bone Marrow Transplantation, Pakistan Kidney and Liver Institute and Research Center, Lahore, Pakistan; 2 Department of Urology, Pakistan Kidney and Liver Institute and Research Centre Lahore, Pakistan

**Keywords:** adrenal tumors, normotensive, pheochromocytoma, adrenalectomy

## Abstract

**Background/Objective:**

Pheochromocytoma is a rare catecholamine-producing tumor that often presents with episodic hypertension, headache, and sympathetic overactivity. Although bilateral adrenal involvement occurs in up to 10% of cases and flank/back pain has been reported in the literature, normotensive presentations without classic symptoms remain diagnostically challenging.

**Case Report:**

We report a 32-year-old woman with bilateral flank pain radiating to the back and no history of hypertension or classical symptoms. Imaging revealed bilateral adrenal masses; hormonal studies showed noticeable increased plasma metanephrine and normetanephrine levels. After preoperative optimization, she underwent a left robotic and right open adrenalectomy. Histopathologic and immunohistochemical analysis confirmed the diagnosis of pheochromocytomas. She was discharged with steroid replacement and remains disease-free on follow-up.

**Discussion:**

This case highlights the atypical presentation of bilateral pheochromocytomas without hypertension. The absence of classic symptoms and normal urinary catecholamines can delay diagnosis. However, increased plasma metanephrine levels and imaging findings were crucial in guiding timely surgical management.

**Conclusion:**

Clinicians should consider pheochromocytoma in patients with unexplained, persistent flank pain—even in the absence of hypertension. Early imaging and plasma metanephrine testing are essential for accurate diagnosis in such atypical cases.

**Highlights**

• Bilateral pheochromocytoma can present without hypertension or classic catecholamine-related symptoms, leading to diagnostic delays
• Normotensive presentation should not exclude pheochromocytoma from the differential diagnosis in patients with adrenal masses or unexplained flank pain
• Advanced imaging and biochemical testing are essential for accurate diagnosis, even in atypical clinical scenarios
• Hybrid surgical approaches (robotic and open adrenalectomy) may be safely employed based on tumor characteristics and surgical access
• Lifelong steroid replacement and multidisciplinary follow-up are critical after bilateral adrenalectomy to ensure optimal recovery and long-term stability

**Clinical Relevance**

This case emphasizes the need to consider pheochromocytoma in normotensive patients with adrenal masses and highlights the importance of personalized surgical planning in managing rare endocrine tumors.

## Introduction

Pheochromocytomas are rare catecholamine-secreting neuroendocrine neoplasms arising from the adrenal gland medullary chromaffin cells.^1^ Their estimated prevalence ranges between 0.1% and 0.6% among hypertensive patients, and they classically present with a triad of paroxysmal headache, palpitations, and diaphoresis, often accompanied by sustained or paroxysmal hypertension.^2^ However, rare presentations can occur in normotensive individuals, often resulting in delayed diagnosis and therapy.^3^

Bilateral pheochromocytomas account for approximate of 10% of all pheochromocytoma cases^4^ and are usually associated with hereditary syndromes such as multiple endocrine neoplasia type 2, von Hippel-Lindau disease, and neurofibromatosis type 1.^4^ However, bilateral pheochromocytoma in the absence of a familial or known genetic predisposition remains relatively uncommon.^5^^,^^6^ In addition, although flank or back pain has been reported as a symptom in the literature,^7^ such presentations in normotensive patients without classic signs of catecholamine excess remain diagnostic challenge and are less recognized as frequent.

Furthermore, the absence of increased 24-hour urinary metanephrine levels, coupled with bilateral tumor localization in a nonsyndromic patient, makes this case an important addition to the spectrum of atypical presentations. We report a case of a 32-year-old normotensive woman with bilateral adrenal masses who presented with sole persistent flank pain radiating to the back. The patient was at first managed symptomatically for musculoskeletal discomfort before being referred to a tertiary care center, where imaging and plasma-based biochemical evaluation led to the diagnosis of bilateral pheochromocytoma.

This case underscores the importance of maintaining a high index of suspicion for pheochromocytoma in patients with unexplained flank or back pain, even in the absence of hypertension or classic symptoms. Early imaging and plasma metanephrine testing can facilitate on time diagnosis and multidisciplinary management.

## Case Report

### Patient Information

A 32-year-old married woman with no significant past medical history presented to the outpatient department of urology at our institute, with complaints of right-sided flank pain radiating to the back. This later progressed to involve the left side. She had 3 children and no family history of endocrine disorders.

### Clinical Findings

Initial presentation included left flank pain (mild to moderate, short-lasting, radiating to the back). Six months later, there was right flank pain (moderate to severe, prolonged, nonresponsive to analgesics). There were no associated symptoms—no headache, palpitations, sweating, nausea, vomiting, urinary complaints, weight loss, or visual changes. The patient was normotensive on multiple visits. Physical examination was unremarkable.

### Timeline

**Table:** 

Time point	Event
2 years ago	Initial mild left flank pain, radiating to the back
18 months ago	Right flank pain, more severe and frequent
Before referral	Multiple physician visits, treated symptomatically
Ultrasound	Bilateral adrenal masses identified
March 4, 2024	Contrast-enhanced computed tomography (CECT) scan performed externally
April 26, 2024	CECT reported: solid/cystic bilateral adrenal lesions
Endocrinology workup	Confirmed pheochromocytoma (plasma metanephrine levels increased)
Positron emission tomography–computed tomography	Bilateral adrenal masses; no metastasis
May 2, 2024	Urology outpatient department visit; surgical planning
May 7, 2024	Preoperative admission and preparation
May 8, 2024	Surgery: left robotic + right open adrenalectomy
May 10, 2024	Discharged on steroid therapy
January 15, 2025	Follow-up CECT: no residual disease

### Diagnostic Assessment

Initial imaging with abdominal ultrasound revealed bilateral adrenal masses, prompting further evaluation. In accordance with the recommended approach to bilateral adrenal masses by Sweeney et al,^7^ the patient underwent a structured diagnostic workup that included detailed imaging (contrast-enhanced computed tomography [CT] and positron emission tomography-CT) and comprehensive biochemical assessment to evaluate hormone hypersecretion, malignancy, and potential hereditary syndromes. This systematic approach helped narrow the differential diagnoses and supported the diagnosis of pheochromocytoma. On CT imaging, pheochromocytomas typically demonstrate an unenhanced attenuation of >10 HU and show avid contrast enhancement with delayed washout, distinguishing them from lipid-rich adenomas, which usually have an attenuation of <10 HU. These imaging characteristics align with criteria outlined by the Endocrine Society and the American College of Radiology Appropriateness Criteria.^8^, ^9^, ^10^

A contrast-enhanced CT scan demonstrated bilateral heterogeneous enhancing solid/cystic adrenal lesions with absolute washout values of <60%, suggestive of pheochromocytoma or atypical adenomas, with a less like differential diagnosis of adrenal metastases from an unknown primary.

Positron emission tomography-CT was performed, revealing bilateral adrenal lesions with increased metabolic activity and no evidence of lymph node, visceral, or bone marrow involvement, suggesting localized adrenal pathology.

A detailed biochemical workup was conducted to assess catecholamine excess and adrenal hormone function (Table 1).

**Table 1:** Biochemical Evaluation of Catecholamine Excess and Adrenal Hormone Function

Test	Patient result	Reference range	Interpretation
Plasma metanephrine	>600 pg/mL	0-62 pg/mL	**Raised**
Plasma normetanephrine	>800 pg/mL	0-145 pg/mL	**Raised**
24-h urinary metanephrine	31.22 μg/d	<350 μg/d	Within normal range
Serum cortisol (morning)	7.8 μg/dL, 5.0 μg/dL	3.70-19.40 μg/dL	Normal
Serum aldosterone	15 ng/dL	4-31 ng/dL (varies a slight by laboratory)	Within normal range
Plasma direct renin	11.09 uIU/mL	2.8-40 uIU/mL	Within normal range
Aldosterone/direct renin ratio	1.35 ng/uIU/mL	<3.7 ng/uIU/mL	Below cutoff for primary aldosteronism
Serum prolactin	3.4 ng/mL	4.8-23.3 ng/mL (female), 4-15.2 ng/mL (male)	**Low**
Serum calcium	Normal	8.6-10.2 mg/dL	Normal
Serum albumin	Normal	3.5-5.0 g/dL	Normal

Serum prolactin was assessed as part of a comprehensive endocrine evaluation. Although not routinely included in all adrenal incidentaloma workups, prolactin testing can help identify possible hypothalamic-pituitary axis involvement or underlying syndromic associations, such as multiple endocrine neoplasia. Given the suspicion of pheochromocytoma, prolactin was measured to rule out coexisting pituitary pathology or syndromic overlap.

Based on biochemical confirmation, the increased plasma metanephrine and normetanephrine levels, the patient was diagnosed with pheochromocytoma.

### Therapeutic Intervention

Before surgery, the patient was initiated on alpha-adrenergic blockade with tablet doxazosin 4 mg twice daily to control potential catecholamine surges. She also underwent medical optimization, including steroid preparation, as recommended by the internal medicine team. Comprehensive preoperative evaluations were carried out, and she received clearance from both the anesthesia and endocrinology departments. The operative procedure comprised a left robotic radical adrenalectomy and a right open radical adrenalectomy for the removal of bilateral adrenal masses. Postoperatively, the patient was admitted to the surgical intensive care unit (SICU), where she was closely monitored and treated with norepinephrine support, which was tapered over time. The steroid replacement therapy was started to replace the adrenal function due to bilateral adrenalectomy.

**Fig. 1:**
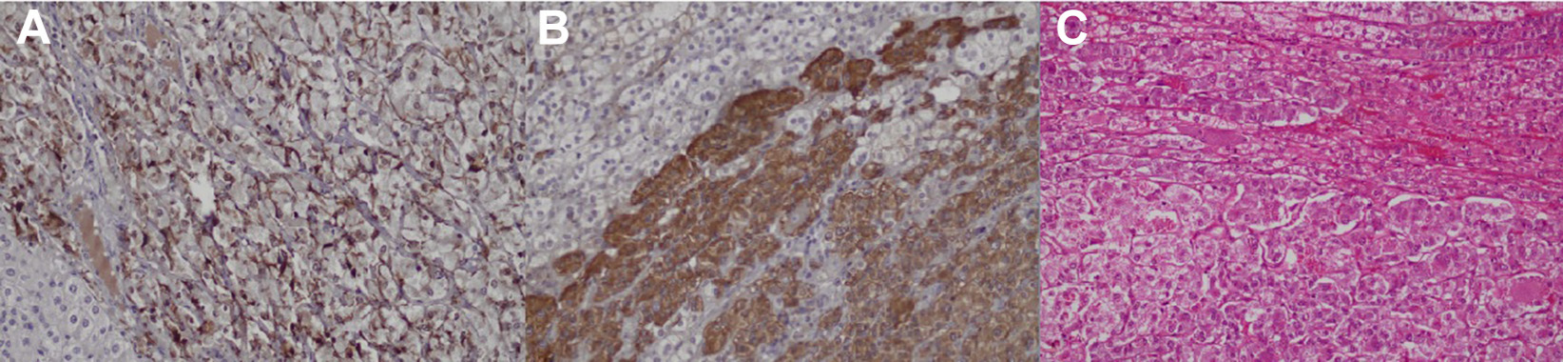
Histopathologic and immunohistochemical features of bilateral adrenal pheochromocytoma. *A*, High power (x20) showed hematoxylin and eosin-stained tissue section with a well-circumscribed tumor composed of a nest of cells arranged in Zell Bellen pattern. The cells had abundant amphophilic cytoplasm. The tumor nests were surrounded by sustentacular cells. The periphery showed a normal adrenal cortex. *B*, Synaptophysin immunohistochemical stain was strongly positive in tumor cells. *C*, S100 immunohistochemical stain highlighted the sustentacular cells.

Biopsy tissues from the 2 adrenal masses were submitted for histopathologic examination to establish the diagnosis. The procedure was completed without intraoperative complications. Histopathologic and immunohistochemical analysis confirmed the diagnosis of pheochromocytoma, as illustrated in Figure 1.

### Follow-Up and Outcomes

Postoperatively, the patient was admitted to the SICU, where she was monitored closely and managed with norepinephrine support, which was gradually tapered. Replacement therapy with steroids was instituted immediately to replace adrenal function after bilateral adrenalectomy. Biopsy of both adrenal masses was taken for histopathologic analysis to secure the diagnosis. The operation was performed safely without any intraoperative complications.

The patient was monitored in the recovery room initially and then transferred to the SICU for observation and postoperative care. In the SICU, the patient was extubated and kept on norepinephrine 0.1 μg/kg/min, which was slowly weaned. Her fluid balance was monitored closely, and she was given intravenous fluids, antibiotics, and analgesics. Investigations of blood during this time were satisfactory. The patient improved clinically and was shifted to the urology ward the following day.

Following a proper evaluation in the ward, the patient was discharged in a stable condition with steroid therapy initiated according to established endocrinology practice guidelines for glucocorticoid replacement after bilateral adrenalectomy.^11^ She was instructed to follow-up in both internal medicine and urology outpatient departments. On follow-up, she had a good recovery. Notably, the patient’s flank pain resolved following bilateral adrenalectomy, supporting a link between the tumor burden and her presenting symptom.

She was prescribed oral hydrocortisone 3 times a day. She was also taught to take another 10 mg of hydrocortisone with the onset of acute illness attacks. The value of lifelong steroid replacement after bilateral adrenalectomy was reinforced, and she was requested to always carry a medical emergency card and have regular follow-ups.

**Fig. 2:**
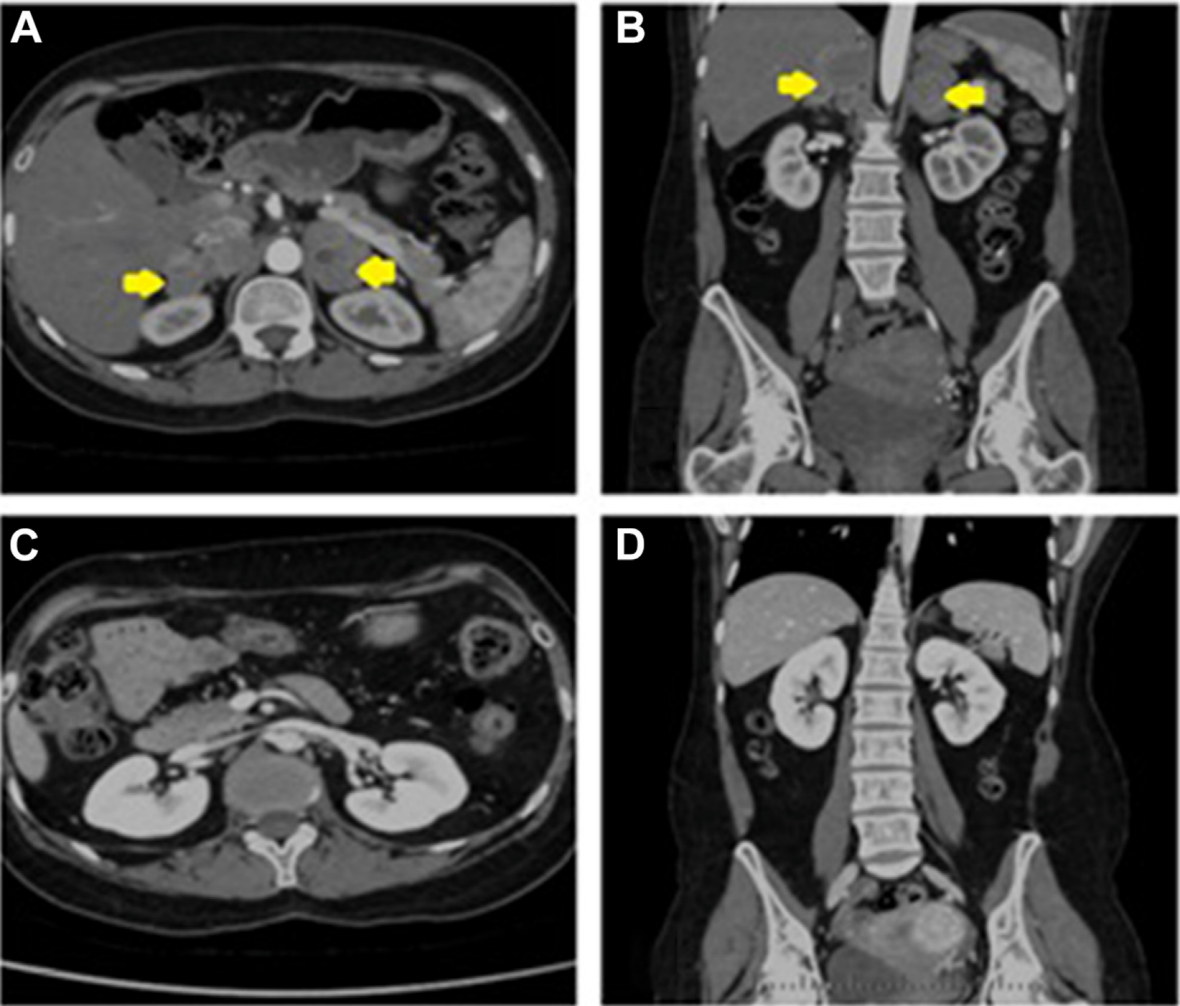
Axial and coronal computed tomography (CT) images demonstrating bilateral adrenal masses before and after adrenalectomy. *A*, Axial view preoperative CT scan. *B*, Coronal view preoperative CT scan (preoperative CT image: yellow arrows showing presence of bilateral pheochromocytoma). *C*, Axial view postoperative scan. *D*, Coronal view postoperative CT scan (postoperative CT images showing absence of bilateral pheochromocytoma following surgical resection).

Contrast-enhanced CT that was performed confirmed no postoperative collections of any significance and no residual disease. The patient remains well under follow-up by the endocrinology and urology teams. Postoperative and preoperative imaging findings are illustrated (Fig. 2).

## Discussion

To our knowledge, this is one of the few reported cases of bilateral pheochromocytoma in a normotensive adult without any identifiable syndromic. The patient presented solely with flank pain, in the absence of classical catecholamine-related symptoms such as hypertension, palpitations, or diaphoresis, leading to a delayed diagnosis. This case highlights the diagnostic and therapeutic challenges associated with atypical, sporadic presentations of bilateral adrenal tumors. Furthermore, the combined use of robotic and open adrenalectomy—tailored to tumor characteristics—offers an important procedural perspective for the surgical management of such rare cases.

Although flank or back pain has been previously documented in patients with pheochromocytoma,^7^ this case is unique due to the bilaterality, absence of hypertension, normal urinary metanephrines, and presentation without any catecholamine-associated symptoms. To our knowledge, few reports exist that combine all these atypical features, making timely diagnosis more difficult and underlining the importance of considering adrenal imaging early in unexplained cases.

Although hypertension has historically been regarded as a hallmark of pheochromocytoma, contemporary clinical experience indicates that many patients especially those diagnosed incidentally or through imaging for unrelated symptoms may present without increased blood pressure. Recent literature supports that normotensive presentations are not uncommon, particularly in localized adrenal pheochromocytomas. This shift emphasizes the importance of maintaining a high index of suspicion even in the absence of classical catecholaminergic symptoms.

In our patient, the persistent bilateral flank pain likely resulted from mass effect and capsular distension of the adrenal glands or irritation of surrounding retroperitoneal structures. This is supported by the complete resolution of pain following bilateral adrenalectomy, reinforcing a causal relationship between tumor presence and symptomatology

Given the patient’s young age and bilateral adrenal involvement, genetic testing is warranted to rule out hereditary syndromes. Although no clinical features suggestive of syndromic association were observed, formal genetic testing is planned as part of long-term follow-up.

Given the rarity and clinical heterogeneity of bilateral pheochromocytoma, particularly in normotensive, nonsyndromic adults, it is essential to evaluate individual cases within a broader clinical context. Published reports provide valuable insight into the spectrum of presentations, underlying etiologies, and treatment outcomes. Although pediatric and syndromic cases are more frequently described, adult presentations without hypertension or identifiable genetic mutations remain underrepresented in the literature. To contextualize our findings, we present a comparative summary of published cases of bilateral pheochromocytoma in Table 2,^11^, ^12^, ^13^, ^14^, ^15^, ^16^, ^17^, ^18^ highlighting key differences in presentation, genetic associations, and management strategies.

**Table 2:** Comparative Summary of Published Cases of Bilateral Pheochromocytoma

Study (author, y)	Age (y)/sex	Presentation	Genetics/syndrome	BP status	Associated findings	Surgery	Outcome
**Current case, 2024**	32/F	Bilateral flank pain, normotensive	None identified	Normotensive	None	Left robotic + right open adrenalectomy	No residual disease; stable on steroids
Khalaf et al,^11^ 2023	14/M	Hypertension, bilateral adrenal + splenic mass	VHL syndrome	Hypertensive	Paraganglioma	Bilateral adrenalectomy	Stable after surgery
Shen et al,^12^ 2023	40/M	Intermittent dizziness, medullary sponge kidney	RET mutation	Variable	Genetic mutation in the son also	Staged laparoscopic adrenalectomy	Good outcome with hormone replacement
Raoudha et al,^13^ 2021	7/M	Fever, abdominal pain, profuse sweating	None reported	Hypertensive crisis	Suspected appendicitis initially	Total + subtotal adrenalectomy	Stable with normal growth at 10-mo follow-up
Lazaar et al,^14^ 2025	28/F	Bilateral vision loss	None identified	Malignant hypertension	Retinal detachment	Unilateral adrenalectomy	Partial visual recovery
Pandya et al,^15^ 2022	12/M	Cardiogenic shock	None reported	Hypertensive	Cardiac dysfunction	Emergency adrenalectomy	Stabilized with support
Khan et al,^16^ 2025	38/F	Flank pain, sweating, palpitations	Genetic testing declined	Hypertensive	None	Left adrenalectomy + right cortical-sparing adrenalectomy	Stable, preserved adrenal function
Madesan and Rajendra,^17^ 2020	24/M	Headache, high BP, papilledema	MEN2/VHL ruled out	Initially hypertensive, later hypotensive	Bilateral papilledema, adrenal vein involvement	Bilateral adrenalectomy	Discharged on antihypertensives and steroids
Zahid et al,^18^ 2024	49/F	Asymptomatic; incidental finding	Tuberous sclerosis complex	Normotensive	Retroperitoneal paraganglioma and ganglioneuroma	Bilateral adrenalectomy + retroperitoneal excision	Normotensive; decreased normetanephrine levels

Abbreviations: BP = blood pressure; F = female; M = male; MEN2 = multiple endocrine neoplasia type 2.

This case underscores several important clinical insights. It expands the limited literature on adult bilateral pheochromocytomas presenting without hypertension or syndromic associations. The integration of advanced imaging, multidisciplinary preoperative optimization, and a hybrid surgical approach (robotic and open) highlights the importance of individualized treatment planning in complex adrenal cases. Although single-case findings are not generalizable, this report reinforces the need to consider pheochromocytoma in the differential diagnosis of adrenal masses, even in normotensive patients lacking classic symptoms or family history. Routine genetic evaluation should also be considered in bilateral cases to guide long-term surveillance and management. Future efforts should aim to increase clinical awareness of atypical pheochromocytoma presentations and encourage a low threshold for biochemical screening in such scenarios.

## Conclusion

Pheochromocytoma can be present without classical features such as hypertension or sympathetic symptoms. Biochemical screening should be considered in patients with unexplained adrenal masses, even if normotensive. Bilateral adrenalectomy requires careful perioperative planning and lifelong steroid replacement. Multidisciplinary care is essential to ensure optimal outcomes in such rare cases.

### Patient Perspective

Flank pain radiating to the back was experienced for several months, initially misattributed to a musculoskeletal cause. Multiple consultations were made; however, a diagnosis was not reached until imaging revealed bilateral adrenal masses. The diagnosis of pheochromocytoma was unexpected and concerning, especially in the absence of classic symptoms. Surgery was advised and successfully performed. Postoperative recovery was smooth, and daily steroid therapy was initiated. Regular follow-up was recommended. Although lifelong medication was initially overwhelming, reassurance and support were provided by the medical team. Gratitude is expressed for timely diagnosis, effective treatment, and improved quality of life.

## Disclosure

The authors have no conflicts of interest to disclose.
